# Identifying Cryptic Mammals With Non‐Invasive Methods: An Effective Molecular Species Identification Tool to Survey Southern African Terrestrial Carnivores

**DOI:** 10.1002/ece3.71223

**Published:** 2025-04-21

**Authors:** Amy Wong, Eduardo Eizirik, Klaus‐Peter Koepfli, Vera de Ferran, Tresia Shihepo, Anna Rose Lay, Julia Zumbroich, Nicola Rooney, Laurie Marker, Anne Schmidt‐Küntzel

**Affiliations:** ^1^ Cheetah Conservation Fund Otjiwarongo Namibia; ^2^ Bristol Veterinary School University of Bristol Langford UK; ^3^ PUCRS, Escola de Ciências da Saúde e da Vida Laboratório de Biologia Genômica e Molecular Porto Alegre Brazil; ^4^ Instituto Pró‐Carnívoros Atibaia Brazil; ^5^ Smithsonian‐Mason School of Conservation George Mason University Front Royal Virginia USA; ^6^ Department of Ecology and Evolutionary Biology University of California Los Angeles California USA; ^7^ ADRx, Inc. Thousand Oaks California USA

**Keywords:** mitochondrial mini‐barcode, Namibia, non‐invasive genetics, scat‐based survey, southern African terrestrial carnivores, thornbush savannah

## Abstract

Carnivores play a vital role in ecosystem health and are thus an important focus for conservation management. Non‐invasive methods have gained traction for carnivore monitoring as carnivores are often elusive and wide‐ranging, making visual counts particularly difficult. Faecal mini‐barcoding combines field collection of scats with genetic analysis for species identification. Here, we assessed the applicability of a mini‐barcode based on the mitochondrial *ATP6* gene in southern Africa. We predicted amplification success based on in silico evaluation of reference sequences from 34 of the 42 terrestrial carnivore species existing in southern Africa, including the Congo clawless otter ( *Aonyx congicus* ) for which we contributed a mitochondrial assembly. We further tested amplification success on available reference samples of 23 species. We expanded the existing *ATP6* mini‐barcode reference database by contributing additional sequences for 22 species, including the Cape genet ( *Genetta tigrina* ) and the side‐striped jackal (*Lupulella adusta*) for which no complete mini‐barcode sequences were available on GenBank, and compiled a representative reference dataset of 61 unique sequences as a tool for species identification. As a proof of principle, we applied the *ATP6* mini‐barcode to a small scat‐based carnivore survey conducted in Namibia 13 years prior, which showed a 95% identification success and detected six species among 157 samples collected. With southern Africa's mammalian carnivores facing escalating threats, this robust mini‐barcode offers a vital tool for accurate species identification from non‐invasive samples, enabling crucial monitoring and conservation efforts.

## Graphical abstract

We evaluated the effectiveness of a mitochondrial mini‐barcode based on the *ATP6* gene to identify terrestrial carnivores in southern Africa, both theoretically and through PCR amplification. We provide a curated reference dataset composed of previously published and newly generated sequences, including three species for which no reference sequence was available for this marker. We applied the mini‐barcode to a field survey in an African thornbush savannah with a 95% identification success.

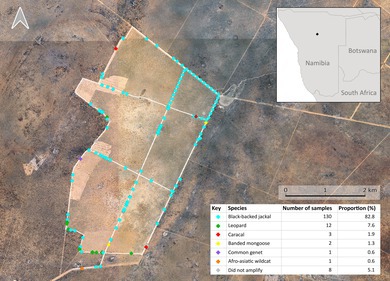

## 1 Introduction

Carnivores are crucial to maintaining intact ecosystem functions, including top‐down trophic cascade effects. They directly influence species abundance in lower trophic levels, consequently also impacting plant communities (Ripple et al. 2014), as well as nutrient cycling within ecosystems by distributing nutrients across landscapes (Schmitz et al. 2010). Mesopredators also affect plant communities through seed dispersal and granivore predation (Botha and le Roux 2022; DeMattia et al. 2004). Despite their ecological importance, carnivores (Mammalia, Carnivora) are one of the most threatened animal groups (Ripple et al. 2014). Most carnivores require large home ranges (Crooks 2002) and are therefore particularly susceptible to habitat depletion and fragmentation (Luyssaert et al. 2014; Ceballos and Ehrlich 2002).

Due to the often elusive, wide‐ranging nature of many carnivore species (Cardillo et al. 2004), traditional survey methods relying on direct observations (e.g., game counts) or animal capture (e.g., radio telemetry) have proven challenging and are less effective than non‐invasive methods based on camera trapping or scat samples (Kelly et al. 2012). Indeed, these non‐invasive methods are more likely to detect species which are present at low densities, as they are based on the accumulation of signs (scat samples and tracks) or extended monitoring (e.g., camera traps). One such method is faecal DNA ‘mini‐barcoding’, as described by Hajibabaei et al. (2006). This involves non‐invasive faeces collection followed by DNA extraction, sequencing, and species identification using reference sequence databases. Mini‐barcodes are short DNA sequences, typically 100–300 bp in length, that are species‐specific and therefore act as markers for species recognition (Hajibabaei et al. 2006).

Faecal mini‐barcoding of mitochondrial DNA (mtDNA) has been shown to be useful for wildlife monitoring as the method is particularly well suited to reliably identify interspecific differences compared to other molecular markers such as microsatellites (Pilot et al. 2006). The short length of mini‐barcodes means they are more likely to be successfully amplified in poor‐quality faecal DNA than the full‐length barcodes (~650 base‐pairs) (Hajibabaei et al. 2006; Meusnier et al. 2008). Therefore, while full‐length barcodes can provide more information, potentially contributing to research in taxonomy, phylogenetics, and population genetics (Hajibabaei et al. 2007), mini‐barcodes are usually the method of choice for species identification in degraded DNA such as preserved tissues, environmental, and faecal DNA (Brinkman et al. 2009; Norris and Michalski 2010; Vynne et al. 2011).

Different mtDNA regions can be targeted for mini‐barcoding. One such mini‐barcode is a 126 base‐pair section of the *ATP synthase 6* (*ATP6*) gene, which was demonstrated to be able to discriminate closely related, recently diverged carnivore species (Chaves et al. 2012; Haag et al. 2009). These *ATP6* primers also did not amplify any prey DNA that could have been present in the faecal sample (Chaves et al. 2012). The amplification success of these *ATP6* primers ranges from 21.2% (Mesa‐Cruz et al. 2016) to 100% (Alberts et al. 2017) and has been demonstrated in carnivores from different habitats, including tropical rainforest (Alberts et al. 2017; Chaves et al. 2012; Gonçalves et al. 2022; Haag et al. 2009; Michalski et al. 2011; Souza et al. 2017; Srbek‐Araujo et al. 2018) and mountainous regions (Mazzolli et al. 2017).

Variability in study success has been attributed to a variety of environmental factors. Samples can be scarce under certain climatic and/or habitat conditions, such as seasonal increases in dung beetle activity (Becker et al. 2017; Norris and Michalski 2010). Environmental exposure may also limit amplification success due to DNA degradation. In tropical climates, samples in open areas showed significantly lower amplification success than those under canopy cover, which was attributed to higher temperatures and UV exposure (Mesa‐Cruz et al. 2016; Michalski et al. 2011). The high humidity of some tropical climates also increases the rate of faecal DNA degradation (Brinkman et al. 2009; Michalski et al. 2011; Vynne et al. 2011). Similarly, the hyper‐arid climate was suggested as the reason for the particularly poor amplification success (25.58%) reported in a study in the South Sinai mountains (Gecchele et al. 2017). Dry samples have been suggested to produce poor success, since dryness is correlated with sample age and thus UV exposure (Michalski et al. 2011; Piggott 2004). The large number of uncontrolled variables and differing study methods highlight the importance of continued research to understand the value of faecal mini‐barcoding in each specific habitat. In particular, savannah and grassland habitats are poorly represented in the literature, and the environmental effects on sample integrity and use of *ATP6* mini‐barcode (Chaves et al. 2012) have not yet been tested in species assemblages in such habitats. Savannah and grasslands are the main habitats of southern Africa and are home to more than 40 carnivore species, many of which are in need of monitoring and conservation action (Guo et al. 2017; Rutherford et al. 2006).

Southern African savannahs are facing threats of agricultural expansion, plantations, urbanisation, and bush encroachment, leading to habitat degradation (Veldman 2016) and consequent biodiversity reduction (Ryan et al. 2016). Despite providing important carnivore habitats and valuable ecosystem services (Carbutt et al. 2017; Scholtz and Twidwell 2022), savannahs and grasslands receive disproportionately little conservation action (Carbutt and Martindale 2014; Wilsey 2021). To make informed conservation decisions about these habitats, it is important to understand the diversity and distribution of the animals occurring there. Therefore, monitoring carnivore populations not only benefits the conservation of these particular species but is also necessary in the context of assessments of habitat health and broader conservation planning of these ecosystems and their biodiversity (Jetz et al. 2019).

Here, we assess the ability of the *ATP6* mini‐barcode primers to amplify and identify the DNA of carnivore species found in southern Africa, using both in silico and laboratory methods. We contribute a previously unavailable mitogenome assembly for a poorly known African carnivore and reference sequences for the mini‐barcode region for 22 other species, and we compile a representative reference dataset for species identification using this marker. We further apply this mini‐barcoding technique to a systematic non‐invasive survey to characterise the carnivore guild present in the Otjozondjupa region of Namibia, an area which exemplifies a thornbush savannah habitat with ongoing bush encroachment, aiming to establish a baseline assessment of the carnivores present in the area and to initiate non‐invasive monitoring of their ecology.

## 2 Materials and Methods

### 2.1 Identification of Southern African Terrestrial Carnivores, Distribution Across Sub‐Saharan Africa, and Available Mitogenomes

A complete list of terrestrial carnivore species occurring in southern African countries, excluding islands (defined as Angola, Botswana, Eswatini, Lesotho, Malawi, Mozambique, Namibia, South Africa, Zambia, and Zimbabwe) was obtained from the International Union for Conservation of Nature (IUCN) Red List of Threatened Species (IUCN 2022). For each species, its presence in each southern African country (Table 1), as well as in other countries from sub‐Saharan Africa (Supp Table S1), was scored to assess the breadth of applicability of the assay.

**TABLE 1:** Distribution of terrestrial carnivores in southern Africa. Species presence as per IUCN Red List for each country (X), and sequence availability (pre‐existing from GenBank or Dryad; novel from this study) are indicated.

Scientific name	Common name	Sequence availability	Angola	Botswana	Eswatini	Lesotho	Malawi	Mozambique	Namibia	South Africa	Zambia	Zimbabwe
*Acinonyx jubatus*	Cheetah	Pre‐existing	X	X	X		X	X	X	X	X	X
*Aonyx capensis*	African clawless otter	Pre‐existing	X	X	X	X	X	X	X		X	X
*Aonyx congicus*	Congo clawless otter	This study (assembly)	X									
*Atilax paludinosus*	Marsh mongoose	Pre‐existing	X	X	X	X	X	X	X	X	X	X
*Bdeogale crassicauda*	Bushy‐tailed mongoose	Missing					X	X			X	X
*Caracal aurata*	African golden cat	Pre‐existing	X									
*Caracal caracal*	Caracal	Pre‐existing	X	X	X	X	X	X	X	X	X	X
*Civettictis civetta*	African civet	Pre‐existing	X	X	X		X	X	X	X	X	X
*Crocuta crocuta*	Spotted hyaena	Pre‐existing	X	X	X		X	X	X	X	X	X
*Crossarchus ansorgei*	Ansorge's cusimanse	missing	X									
*Cynictis penicillata*	Yellow mongoose	Pre‐existing	X	X		X			X	X		X
*Felis lybica*	Afro‐asiatic wildcat	Pre‐existing	X	X	X	X	X	X	X	X	X	X
*Felis nigripes*	black‐footed cat	pre‐existing	X	X	X	X			X	X		X
*Genetta angolensis*	Miombo genet	Missing	X				X	X			X	
*Genetta genetta*	Common genet	Pre‐existing	X	X		X		X	X	X	X	X
*Genetta maculata*	Rusty‐spotted genet	Missing	X			X	X	X	X	X	X	X
*Genetta tigrina*	Cape genet	This study (barcode)				X				X		
*Helogale parvula*	Common dwarf mongoose	Pre‐existing	X	X	X		X	X	X	X	X	
*Herpestes flavescens*	Kaokoveld slender mongoose	Missing	X						X			
*Herpestes ichneumon*	Egyptian mongoose	Pre‐existing	X	X	X		X	X	X	X	X	X
*Herpestes pulverulentus*	Cape grey mongoose	Missing				X			X	X		
*Herpestes sanguineus*	Common slender mongoose	Pre‐existing	X	X	X		X	X	X	X	X	X
*Hydrictis maculicollis*	Spotted‐necked otter	Pre‐existing	X	X		X	X	X	X	X	X	X
*Ichneumia albicauda*	White‐tailed mongoose	Pre‐existing	X	X	X	X	X	X	X	X	X	X
*Ictonyx striatus*	Zorilla	Pre‐existing	X	X	X	X	X	X	X	X	X	X
*Leptailurus serval*	Serval	Pre‐existing	X	X	X	X	X	X	X	X	X	X
*Lupulella adusta*	Side‐striped jackal	Pre‐existing/this study^a^	X	X	X		X	X	X	X	X	X
*Lupulella mesomelas*	Black‐backed jackal	Pre‐existing	X	X	X	X		X	X	X		X
*Lycaon pictus*	African wild dog	Pre‐existing	X	X	X		X	X	X	X	X	X
*Mellivora capensis*	Honey badger	Pre‐existing	X	X	X		X	X	X	X	X	X
*Mungos mungo*	banded mongoose	pre‐existing	X	X	X		X	X	X	X	X	X
*Nandinia binotata*	African palm civet	pre‐existing	X				X	X			X	X
*Otocyon megalotis*	Bat‐eared fox	Pre‐existing	X	X				X	X	X		X
*Panthera leo*	Lion	Pre‐existing	X	X	X	X	X	X	X	X	X	X
*Panthera pardus*	Leopard	Pre‐existing	X	X	X	X	X	X	X	X	X	X
*Paracynictis selousi*	Selous's mongoose	Missing	X	X			X	X	X	X	X	X
*Parahyaena brunnea*	Brown hyaena	Pre‐existing	X	X	X			X	X	X		X
*Poecilogale albinucha*	African striped weasel	Pre‐existing	X	X	X	X	X	X	X	X	X	X
*Proteles cristata*	Aardwolf	Pre‐existing	X	X	X	X		X	X	X	X	X
*Rhynchogale melleri*	Meller's mongoose	Pre‐existing			X		X	X		X	X	X
*Suricata suricatta*	Meerkat	Pre‐existing	X	X					X	X		
*Vulpes chama*	Cape fox	Pre‐existing	X	X	X	X			X	X		

^a^ The available GenBank sequence was used to assess primer mismatches as the primer binding site was available, but not for sequence comparison, as 59 of the 126 bp mini‐barcode were missing.

Reference mitochondrial sequences containing the *ATP6* gene were obtained from the NCBI GenBank sequence repository (Clark et al. 2016) using the search terms “[scientific species name]” AND “atp6” in the NCBI nucleotide search algorithm of Geneious Prime 2023.1.1 (https://www.geneious.com). The last search was performed on 27 December 2023. Reviewed GenBank entries ('NC_XXXXXX') were omitted and only their original submissions included. Historical sequences (from specimens which were sampled outside the extant continental range, or identified as cave findings, or extinct) were also removed. Sequences of individuals occurring outside of Africa, in captivity, or of unknown origin, were only included if no other reference sequence was available for that species. An additional species could be assessed, as a novel mitogenome sequence assembly was obtained for the Congo clawless otter ( *Aonyx congicus* ) as part of this study (see below) and was included with the downloaded sequences. Finally, only terrestrial carnivore species were considered; therefore, the Cape fur seal ( *Arctocephalus pusillus* ) and southern elephant seal ( *Mirounga leonina* ), two pinniped species that occur in southern Africa, were not included in the analysis. See Supp Table S2 for an overview of all included sequences.

### 2.2 In Silico Assessment of the *ATP6* Mini‐Barcode Primers

All available mitogenomes were truncated to 172 bp corresponding to the *ATP6* amplicon (including primer target sequences) and aligned with the Geneious Alignment tool using default settings in Geneious Prime 2023.1.1 (https://www.geneious.com). For each species, only one representative sequence was kept for each unique sequence variant to facilitate the identification of mismatches in the primer binding sites. Accession numbers of unique sequences used for the assessment are indicated in Supp Table S3. Primer‐template mismatches to the ATP6‐DF3 (AACGAAAATCTATTCGCCTCT) and ATP6‐DR1 (CCAGTATTTGTTTTGATGTTAGTTG) primers (Haag et al. 2009) were recorded and presented in table format. We further evaluated the primer‐template mismatches to assess potential limitations for successful amplification across the target species. For graphical representation of expected amplification success per southern African country, we classified species with 0–1 mismatch in the 5 most 3' nucleotides of the primer binding site as “assumed successful” and species with 2 mismatches as “potentially problematic”.

### 2.3 Verification of Amplification Success of the *ATP6* Mini‐Barcode

In addition to the in silico approach, we assessed empirical PCR amplification and sequencing success for all the species of southern African terrestrial carnivores for which reference samples were available at the genetic laboratories of the Cheetah Conservation Fund (CCF; Otjiwarongo, Namibia) and the Pontifical Catholic University of Rio Grande do Sul (PUCRS; Porto Alegre, Brazil). Extraction, amplification, and sequencing results of 15 species at PUCRS were previously reported by Chaves et al. (2012). Extractions of frozen blood or tissue samples for 17 Namibian species available at CCF were carried out as part of this study, using the Qiagen DNeasy Blood and Tissue Kit according to the manufacturer's protocol. DNA quantity and quality were assessed with gel electrophoresis and DNAs were diluted accordingly. PCR amplification was performed with the ATP6‐DF3 and ATP6‐DR1 primers (0.4 μM final concentration) in 10 μL reactions using AmpliTaq Gold 360 Master Mix (Applied Biosystems) with a touch‐down PCR (decreasing annealing temperature by 1°C, from 60°C to 50°C over the course of the first 10 cycles) in a MiniAmp Thermal Cycler (Applied Biosystems). DNA amplification was verified with gel electrophoresis, and successful amplification recorded as part of the primer assessment. Successfully amplified PCR products were cleaned with ExoSAP‐IT (Applied Biosystems) and sequenced with the Sanger method, which provided additional reference sequences for the mini‐barcode (see below, ‘Building an *ATP6* sequence reference database’).

### 2.4 Building a Reference Database for the *ATP6* Mini‐Barcode

We used several approaches to construct a reference database of *ATP6* gene sequences for southern African terrestrial carnivores that could be used to identify species with the 126 bp sequence (172 bp amplicon) *ATP6* mini‐barcode. We included all available GenBank sequences which had been used for the in silico primer assessment, with the exception of the side‐striped jackal (*Lupulella adusta*, reported as *Canis adustus* , KT448271) which was missing 59 nucleotides for the mini‐barcode. In addition to sequences available on Genbank (see above, ‘In silico assessment of *ATP6* mini‐barcode primers’), we included 10 sequences previously published by Chaves et al. (2012) and deposited on DRYAD (doi: 10.5061/dryad.75748) as well as additional sequences generated as part of this study (Supp Table S2).

We generated additional *ATP6* reference sequences for the 17 species for which Namibian reference samples were available at CCF's Conservation Genetics Laboratory (Otjiwarongo, Namibia) to contribute regional reference sequences for southern Africa. Sequences were obtained with primers ATP6‐DF3 and ATP6‐DR1 from the PCR products that had been obtained as part of the marker assessment (see above, ‘Verification of amplification success of the *ATP6* mini‐barcode’). Furthermore, we generated *ATP6* reference sequences from eight species and one additional subspecies at the University of California, Los Angeles (California, USA) to either fill in species lacking sequences for the mini‐barcode on Genbank (Cape genet, *Genetta tigrina* , and side‐striped jackal) or to augment geographic and possible haplotypic coverage. Sequences were obtained using primers ATP6‐DF1 and ATP6‐DR1 (Trigo et al. 2008). Details of the laboratory work are found in Supp File S4. All resulting sequences were used for sequence comparisons.

We also generated a new mitochondrial genome assembly for the Congo clawless otter ( *Aonyx congicus* ), which ranges into northern Angola, using previously reported short reads of *Aonyx congicus* (NCBI SRX15437983; de Ferran et al. 2022). We used the PALEOMIX pipeline version 1.2.13.251 (Schubert et al. 2014) to filter reads shorter than 18 bp and with quality scores lower than 30 and map the remaining reads against a mixed reference which included the closest available nuclear (Asian small‐clawed otter, *Amblonyx cinereus* , DNA Zoo's Hi‐C assembly) and mitochondrial (African clawless otter, *Aonyx capensis* , NCBI NC046484) references, using the BWA‐backtrack algorithm (Li and Durbin 2009). We extracted the focal segment of the *ATP6* gene from this mitogenome and included it in our database of reference *ATP6* sequences for both the in silico marker evaluation and sequence comparisons.

We created a final reference alignment of the *ATP6* mini‐barcode by integrating the GenBank sequences with the newly generated sequence data and employing the Geneious Alignment tool using default settings in Geneious Prime 2023.1.1 (https://www.geneious.com) and the ClustalW algorithm within the MEGA 11.0.13 package (Tamura et al. 2021). We used this dataset to directly compare between‐ vs. within‐species variation (when more than one sequence per species was available) in this segment and to assess how many base‐pair differences could be used as a threshold for species identification. As a complementary approach, we also performed phylogenetic analyses of this dataset using distance‐based and maximum‐likelihood (ML) approaches. The aim of these phylogenetic analyses was to assess species‐level monophyly (when more than one sequence was available for a given species) with this mini‐barcode, which provides a useful approach to measure this marker's performance for species identification. For the distance‐based methods, we used the neighbour‐joining algorithm implemented in MEGA to compare the diagnostic performance (i.e., species‐level monophyly and its bootstrap support value) of a simple p‐distance versus best‐fit probabilistic substitution models estimated from the dataset (using the ML‐based model selection tool in MEGA). Nodal support was assessed with 500 nonparametric bootstrap replicates. For the ML analysis, we employed IQ‐TREE 2.3.4 (Nguyen et al. 2015) with the best‐fitting model of nucleotide substitution (HKY with gamma correction (alpha = 0.3)) as estimated in MEGA. Nodal support was assessed with 1000 ultrafast bootstrap replicates (Hoang et al. 2018).

### 2.5 Applying the *ATP6* Mini‐Barcode to Field‐Collected Faecal Samples: A Namibian Case Study

The non‐invasive carnivore survey was conducted in 2009, on farm Elandsvreugde (20°25′S, 17°4′E) of CCF near Otjiwarongo (Namibia), in an area known as the ‘big field’, characterised as open grassland at the time of the study. Sample collection was performed as part of ongoing research at CCF and was covered by the sample collecting permit 1385/2009. The total study area covered 14.92 km^2^ of uncultivated land at 1520–1550 m above sea level. Potential carnivore species present in the area included: cheetah ( *Acinonyx jubatus* ), leopard ( *Panthera pardus* ), caracal ( *Caracal caracal* ), Afro‐asiatic wildcat ( *Felis lybica* ), black‐backed jackal (*Lupulella mesomelas*), bat‐eared fox ( *Otocyon megalotis* ), brown hyaena ( *Parahyaena brunnea* ), aardwolf ( *Proteles cristata* ), and common genet ( *Genetta genetta* ) (EIS Namibia 2022).

Existing roads were used as transects, and transect length totalled 30.5 km. Four roads demarcated the perimeter of the study area and separated the open grassland from surrounding bush, and the open study area itself was separated into six parts by one longitudinal and two transversal roads. Transects were covered within 2 h by six groups of one or two individuals simultaneously, searching for scat samples by walking along the road, in May 2009. There was an average high temperature of 25°C and low temperature of 12°C, 0 mm of precipitation and an average humidity of 40% (Weather Underground 2022). Samples were collected using clean sticks or twigs found in proximity of the sample to push the sample into a clean grip seal bag, and the location was recorded using a global positioning system (GPS). Samples were collected regardless of physical status (fresh, dry, and starting to decay), as long as faecal material remained visible. All samples were inventoried and placed into –20°C freezers within 3 h of collection and kept frozen for 13 years, until analysis.

**FIGURE 1:**
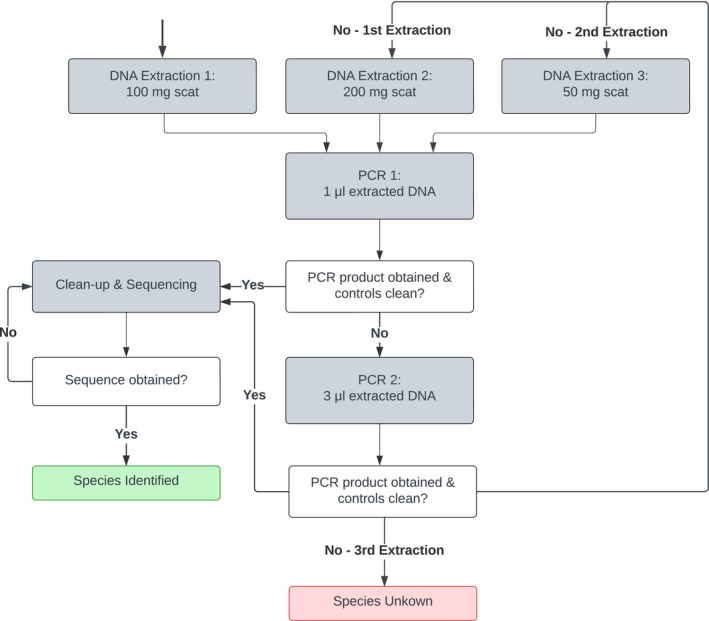
Laboratory workflow for carnivore species identification from field‐collected scat samples, using *ATP6* mini‐barcoding and up to three extractions per sample.

All samples for the survey were processed at the CCF Conservation Genetics Laboratory (Otjiwarongo, Namibia). Extractions were performed in a dedicated non‐invasive room, using Qiagen QIAamp DNA Stool Mini Kits with the following modification to the manufacturer's protocol: varying amounts of faecal material were used (detail hereafter and Figure 1), and elution was performed with only 100 μL to improve DNA concentration. Initial DNA extraction was performed with reduced starting material (100 mg of scat instead of 200 mg in the manufacturer's protocol) to compensate for the weight loss from desiccation in field‐collected samples and because improved amplification success was achieved with 100 mg for a majority of samples in a comparable set of field‐collected samples (data not shown). If a second and possibly third extraction were needed, sample amounts were changed to 200 mg (as per manufacturer's protocol) and 50 mg, respectively, to account for the variability between samples (Figure 1). Note that not all samples required more than one extraction. One negative control (absence of sample) was included in each extraction batch of 3–11 samples. DNA was stored at 4°C until amplification. DNA amplification was carried out in 10 μL reactions, with ATP6‐DF3 and ATP6‐DR1 primers (0.4 μM final concentration), bovine serum albumin (0.4 mg/mL final concentration; Cypress Diagnostics), and varying amounts of DNA (1 μL for PCR 1, and if needed, 3 μL for PCR 2; Figure 1), using AmpliTaq Gold 360 Master Mix (Applied Biosystems). The same touch‐down PCR conditions as for the blood and tissue samples were used for the faecal samples. Extraction controls were included in PCRs alongside negative and positive PCR controls. Successfully amplified PCR products (and any negative extraction or PCR control that may amplify) were cleaned and sequenced as described for tissue reference samples (see above). If amplification failed for both PCRs of all three extractions, the sample was classified as ‘failed to amplify’ and the species recorded as unknown (Figure 1).

Alignments to our curated reference dataset were carried out with the Geneious Alignment tool using default settings in Geneious Prime 2022.1 (https://www.geneious.com), and species identification was performed with two approaches: (i) direct assessment of sequence identity and (ii) phylogenetic analysis. For the identity comparison, a maximum of two mismatches relative to reference sequences from the same area were considered acceptable for species assignment. For the phylogenetic analysis, we performed a neighbour‐joining search with p‐distances and 500 bootstrap replicates to assess nodal support.

Sample locations were mapped using QGIS software version 3.26.1‐Buenos Aires. Samples with missing GPS coordinates were distributed evenly along the road on which they were collected, between known GPS coordinates from the samples collected before and after. Species incidence data were used to plot both a sample‐size‐based rarefaction and extrapolation sampling curve and a sample completeness curve using the iNEXT online software (Chao et al. 2016). We performed a Chi^2^ goodness‐of‐fit test to verify whether black‐backed jackal scat was distributed as expected relative to the length of road running in edge habitat, at the boundary of the bush and open grassland, compared to roads located within open grassland. We performed a Fisher's exact test to determine whether jackal scat distribution differed from other carnivores. Analysis was carried out both with and without the samples missing GPS points.

## 3 Results

### 3.1 Terrestrial Southern African Carnivores

**FIGURE 2:**
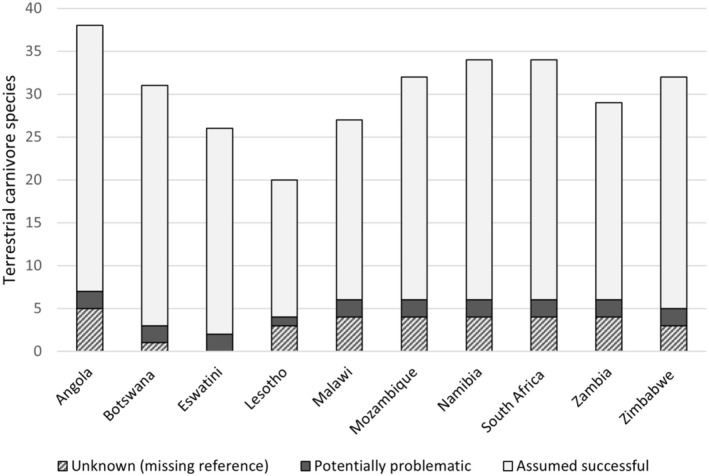
Number of terrestrial carnivore species in each southern African country, with indication of predicted amplification success, when known. Predicted amplification success was based on primer‐template mismatches in the five nucleotides of the 3′ end of each primer, and qualified as ‘assumed successful’ for 0–1 mismatch, or ‘potentially problematic’ for 2 mismatches within the same primer (details in Table 2).

Forty‐two terrestrial species of carnivores occur in mainland southern Africa as per the IUCN Red List (IUCN 2022). Each southern African country listed from 20 (Lesotho) to 38 (Angola) of those terrestrial carnivores as extant (Figure 2; Table 1). Of the 42 species, 9 (Equatorial Guinea) to 27 (Uganda) were also present in other sub‐Saharan countries (Supp Table S1).

We obtained 117 mitochondrial sequences from GenBank for 33 of the 42 extant southern African terrestrial carnivore species (Appendix S2). As part of this study, a 118^th^ sequence was added to GenBank for a 34^th^ species, the Congo clawless otter. Each southern African country included from 17 (Lesotho) to 33 (Angola) of the 34 species for which sequences are available (Table 1, Figure 2). Four sequences were not included because (1) the associated publication was withdrawn (KF907306), (2) the coverage of the amplicon was insufficient (MK469963 and KR132591), or (3) the sequence included ambiguities that were represented clearly in other sequences of the same species (MG772937). Sequences of non‐African, captive, or uncertain origin were only included if no sequences identified as being of African origin were available, leaving a total of 90 sequences, of which 46 were unique (Supp Table S2).

### 3.2 In Silico Assessment of the *ATP6* Mini‐Barcode Primers

Primer‐template mismatches in the primer binding sites of the 34 reference species ranged from 0 to 6 nucleotides, most of which were found in the primer binding site of the ATP6‐DR1 reverse primer (Supp Table S3). When focusing on the five nucleotides at the 3′ end of the primer binding sites, which are the most sensitive to mismatches (Stadhouders et al. 2010), only 0–2 mismatches were identified per species (Table 2). No mismatches were identified in this stretch of the 3′ end of the primer sequence for 19 species, while a single primer‐template mismatch was identified in the forward primer of one species and in the reverse primer of 12 species, and two primer‐template mismatches were identified in the 3′ end of the reverse primer of two species: African wild dog ( *Lycaon pictus* ) and white‐tailed mongoose ( *Ichneumia albicauda* ) (Table 2). African wild dog had a T‐G mismatch in the third position and a G‐T mismatch in the fourth position. White‐tailed mongoose had a T‐G mismatch in the second position and a G‐T mismatch in the fourth position (Table 2). All primer‐template mismatches identified in the 3′ ends of the primers, including those of the two species with two mismatches, were described as ‘Acceptable’ by Stadhouders et al. (2010). The proximity of the two mismatches to each other could however compound the issue; therefore, we qualified those two species as ‘potentially problematic’ for successful amplification in Figure 2.

**TABLE 2:** Predicted and empirical amplification success of the *ATP6* mini‐barcode in southern African terrestrial carnivore species.

Scientific name	Common name	ATP6‐DF3 3′ end					Total mismatches			ATP6‐DR1 3′ end					Amplified
	Amplicon position	17	18	19	20	21	(full primer length)			148	149	150	151	152	Reference
	Primer nucleotide	C	C	T	C	T	F	Cumulative	R	G	T	T	G	A	Samples
*Acinonyx jubatus*	Cheetah						0	2	2						Yes
*Aonyx capensis*	African clawless otter						3	5	2						Not tried
*Aonyx congicus*	Congo clawless otter						3	5	2						Not tried
*Atilax paludinosus*	Marsh mongoose						1	5	4				G‐T		Not tried
*Bdeogale crassicauda*	Bushy‐tailed mongoose														Not tried
*Caracal aurata*	African golden cat						1	4	3				G‐T		Not tried
*Caracal caracal*	Caracal						1	2	1						Yes
*Civettictis civetta*	African civet						0	5	5						Yes
*Crocuta crocuta*	Spotted hyaena						1	4	3						Yes
*Crossarchus ansorgei*	Ansorge's cusimanse														Not tried
*Cynictis penicillata*	Yellow mongoose						0	1	1						Not tried
*Felis lybica*	Afro‐asiatic wildcat						0	1	1				G‐T		Yes
*Felis nigripes*	Black‐footed cat						0	1	1						Yes
*Genetta angolensis*	Miombo genet														Not tried
*Genetta genetta*	Common genet						1	5	4						Yes
*Genetta maculata*	Rusty‐spotted genet														Not tried
*Genetta tigrina*	Cape genet														Not tried
*Helogale parvula*	Common dwarf mongoose						1	5	4		T‐G				Yes
*Herpestes flavescens*	Kaokoveld slender mongoose														Not tried
*Herpestes ichneumon*	Egyptian mongoose						0	3	3				G‐T		Not tried
*Herpestes pulverulentus*	Cape grey mongoose														Not tried
*Herpestes sanguineus*	Common slender mongoose						0	2	2				G‐T		Yes
*Hydrictis maculicollis*	Spotted‐necked otter						2	5	3						Not tried
*Ichneumia albicauda*	White‐tailed mongoose						0	6	6		T‐G		G‐T		Yes
*Ictonyx striatus*	Zorilla						1	6	5				G‐T		Yes
*Leptailurus serval*	Serval						0	1	1				G‐T		Yes
*Lupulella adusta*	Side‐striped jackal						1	4	3				G‐T		Not tried
*Lupulella mesomelas*	Black‐backed jackal						1	4	3				G‐T		Yes
*Lycaon pictus*	African wild dog						0	5	5			T‐G	G‐T		Yes
*Mellivora capensis*	Honey badger						3	7	4						Not tried
*Mungos mungo*	Banded mongoose						0	4	4				G‐T		Yes
*Nandinia binotata*	African palm civet						1	3	2						Not tried
*Otocyon megalotis*	Bat‐eared fox						1	6	5						Yes
*Panthera leo*	Lion						1	2	1						Yes
*Panthera pardus*	Leopard						1	2	1						Yes
*Paracynictis selousi*	Selous's mongoose														Not tried
*Parahyaena brunnea*	Brown hyaena						1	3	2						Yes
*Poecilogale albinucha*	African striped weasel						1	7	6						Not tried
*Proteles cristata*	Aardwolf						1	5	4						Yes
*Rhynchogale melleri*	Meller's mongoose						1	5	4				G‐T		Yes
*Suricata suricatta*	Meerkat			T‐G			2	5	3						Yes
*Vulpes chama*	Cape fox						0	5	5						Yes

*Note:* Primer‐template mismatches for the 3′ ends of both primers (forward F in 5′–3′ orientation and reverse R in 3′–5′ orientation) relative to available GenBank sequences, as well as total mismatches for the entire primer sequences and cumulative mismatches for the primer pair are indicated. In the absence of GenBank sequence, the fields are greyed out. Successful amplification of available reference samples is indicated in the last column. In the absence of sample, this is indicated as ‘not tried’. For Accession numbers of GenBank sequences used to predict primer‐template mismatches and detail of primer‐template mismatches for the full primer sequence, see Appendix S3.

### 3.3 Amplification Success of the *ATP6* Mini‐Barcode

For 23 of the southern African terrestrial carnivore species, primer amplification was successfully verified in blood and tissue reference samples, including for the African wild dog and the white‐tailed mongoose, the two species for which amplification was predicted to be potentially problematic based on the 5 most 3' nucleotides (Table 2, Supp Table S3).

### 3.4 Reference Database for the *ATP6* Mini‐Barcode

*ATP6* mini‐barcode reference sequences were generated for 22 species as part of this study, including 17 species for which amplicons had been generated to verify amplification success and eight species (three of which in common with the aforementioned set) for which sequences had been generated for species of interest with a fragment also including *ATP8*. The newly generated reference sequences included the Cape genet and the side‐striped jackal, for which no or only an incomplete ATP6 reference sequence was previously available on GenBank. Thereby we were able to expand the number of reference species available for the mini‐barcode to the scientific community by two species. For the remaining 20 species, published reference sequences were available on GenBank already and differed from the new reference sequences by 0–10 bp.

Furthermore, we generated a 16,188 bp assembly of the mitochondrial genome of the Congo clawless otter with a coverage of 105× (PV256478, doi: 10.5061/dryad.bcc2fqzmt), adding a new reference sequence for a third species to GenBank.

Our final curated dataset contained 137 sequences representing 35 species (Supp Table S3 and Supp Figure S5). Some species were represented by a single individual (whose sequence in every case was distinct from sequences sampled in other species), while others were represented by two or more individuals. The latter case included species whose sampled sequences were all identical and others which exhibited some intraspecific variation. From the full curated alignment, we selected a representative reference dataset of 61 sequences, including all unique haplotypes. This representative dataset is available on Dryad to be used as a species identification tool (doi: 10.5061/dryad.bcc2fqzmt) and will be updated periodically as additional species are sampled and/or new sequence variants are identified.

Phylogenetic analyses with all distance‐based and maximum likelihood approaches led to consistent results in terms of species‐level monophyly, with minor differences among methods (Supp Figures S6 and S7). Only species‐level nodes were assessed for this purpose since the goal of the analyses was not to reconstruct relationships among species. The simplest method (neighbour‐joining with p‐distances) presented the best diagnostic performance, with all the species represented by more than one sequence being retrieved as monophyletic, in most cases with high bootstrap support (Supp Figure S6). This result is likely due to the short sequences in the mini‐barcode containing too few characters for accurate estimation of the probabilistic DNA substitution model, in which case the use of a simple, empirical distance (*p*‐distance) is more justified.

### 3.5 Applying the *ATP6* Mini‐Barcode to Field‐Collected Samples: A Namibian Case Study

**FIGURE 3:**
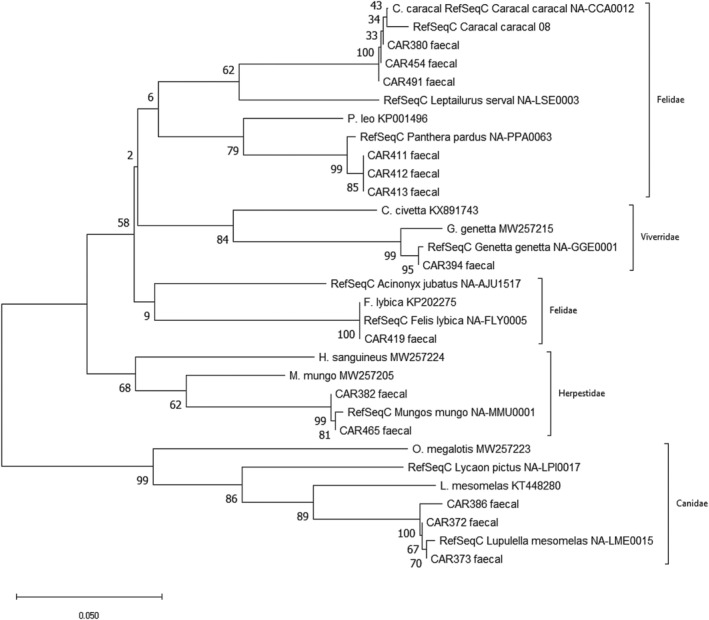
Phylogenetic relationships among *ATP6* mini‐barcode sequences derived from field‐collected faecal samples (labelled ‘CARXXX faecal’) and reference sequences from our curated dataset (Supp Table S2). Only reference sequences with the highest similarity to the faecal samples are included, along with selected sequences from closely related species for comparison. The phylogeny was estimated using the neighbour‐joining algorithm and *p*‐distances, with nodal support assessed with 500 nonparametric bootstrap replicates. Only nodes testing species‐level monophyly were used to assess the performance of the marker.

Of the 157 collected faecal samples, species identity could be assigned to 94.9% (*n* = 149). Moreover, 72.6% of the samples (*n* = 114) successfully amplified on the first attempt (Table 3). From the 149 successfully amplified samples, six species were identified: black‐backed jackal (*n* = 130), leopard (*n* = 12), caracal (*n* = 3), Afro‐asiatic wildcat (*n* = 1), common genet (*n* = 1), and banded mongoose ( *Mungos mungo* ; *n* = 2) (Figure 3). The banded mongoose could only be reliably identified using the newly obtained reference mini‐barcode sequence, which it matched with 100% sequence identity. The majority of samples were identified as black‐backed jackal (*n* = 130), while the smallest proportion of samples was identified as common genet and Afro‐asiatic wildcat (*n* = 1 each).

**TABLE 3:** Number of amplification attempts required for faecal DNA from field‐collected scat samples to obtain a sequence allowing carnivore species identification.

Attempt	Number of samples attempted	Number of samples assigned to species	Percentage assigned to species/failed (%)
Extraction 1, PCR 1	157	114	72.6
Extraction 1, PCR 2	43	21	13.4
Extraction 2, PCR 1	22	11	7.0
Extraction 2, PCR 2	11	3	1.9
Extraction 3, PCR 1	8	0	0
Extraction 3, PCR 2	8	0	0
Failed To Amplify	8	n/a	5.1

**FIGURE 4:**
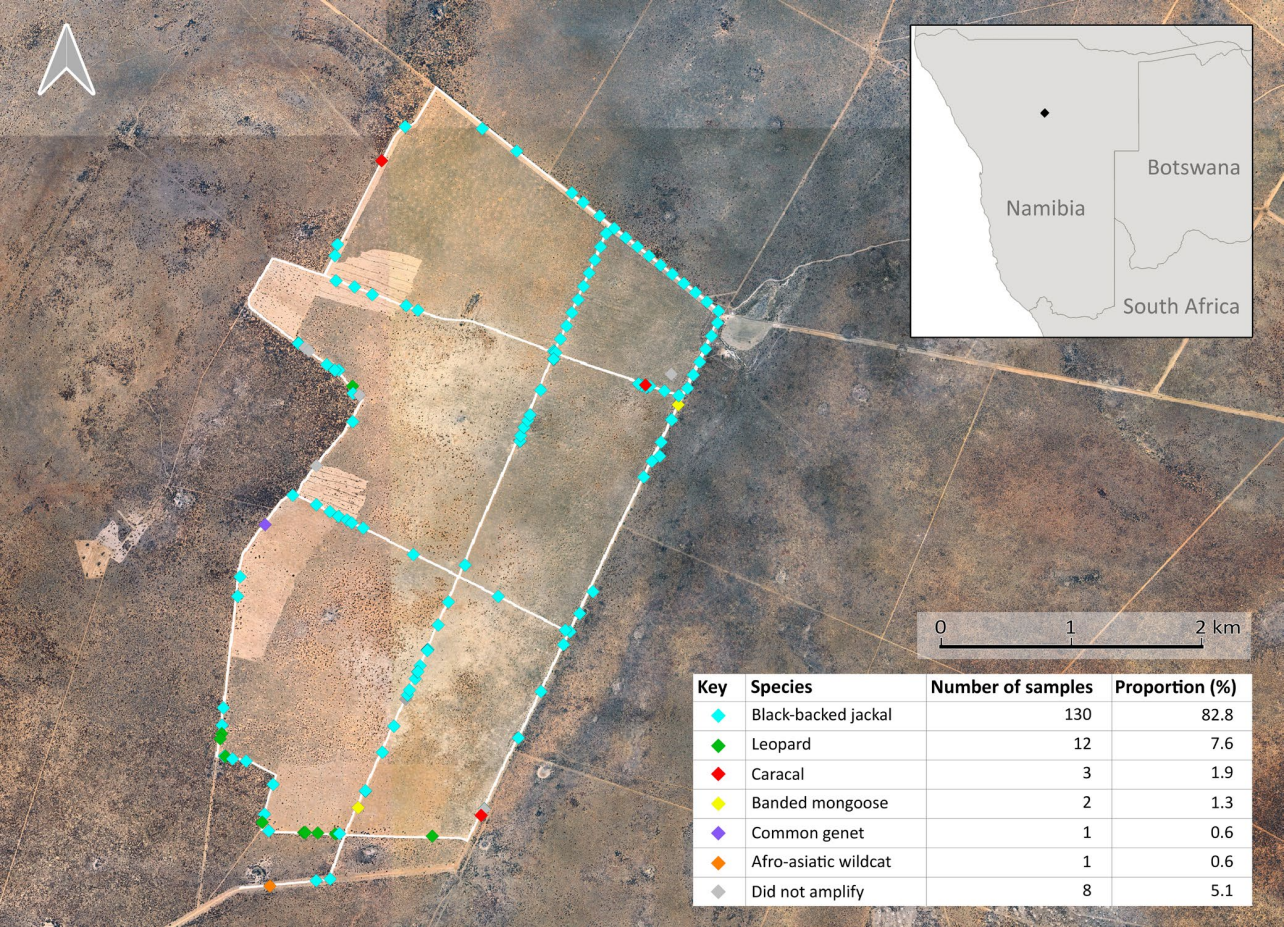
Distribution* of carnivore scat samples between open and edge (bush) habitat, in the study area, located in the Otjozondjupa region of Namibia. Species identity obtained with *ATP6* mini‐barcoding was identified by colour code as shown in the legend. *Scat locations in the top right quadrant were approximated due to missing GPS coordinates; they were placed by evenly spacing them out between known GPS coordinates.

The sample completeness curve suggests that a sample size of 149 (as identified here) describes the carnivores of the study area to 98.67% and that nine samples are sufficient to account for 92% sample coverage (Supp Figure S8A). However, we note that black‐backed jackals account for 87% of the entire sample collection, so that 92% sample coverage would still be likely to miss the majority of other species. Supp Figure S8B indicates that 500–600 samples would be required to detect full species assemblage in the study area and suggests that the sample size of this study may have led to two species likely being missed. Jackal scat was evenly distributed between border roads and non‐border roads, as confirmed statistically with a Chi^2^ goodness‐of‐fit test (χ^2^ = 1.673, *p*‐value = 0.19582 at *p* < 0.05; values corresponding to analysis without estimated GPS locations; similar values were obtained when including the estimated GPS locations; Figure 4). The distribution of black‐backed jackal scat differed significantly from other carnivores, which were all found on border roads (Fisher exact statistic value = 0.00001; *p* < 0.01; Figure 4).

## 4 Discussion

Our study demonstrates that the published *ATP6* mini‐barcode is a highly effective tool for the identification of terrestrial carnivore species in southern Africa and suggests similar utility for other African countries. This mini‐barcode is efficient both in terms of its ability to detect existing carnivore species as well as its applicability for field surveys.

The *ATP6* mini‐barcode was predicted to successfully amplify with currently available primers in 93% of assessed reference sequences (*N* = 34 species). It is expected to reliably detect 91%–94% of reported terrestrial carnivore species in southern African countries as per the evaluated reference sequences (which, after addition of the Congo clawless otter, were available for 85%–100% of all reported terrestrial carnivore species in those countries), making it a valuable tool for conservation research. These same species are also widely distributed throughout other sub‐Saharan countries, so these primers are expected to successfully amplify in 88%–100% of evaluated species in those countries (% of available species was not assessed for other sub‐Saharan countries, as only southern African species were considered for this study), supporting the broad applicability of the marker. Actual species detectability may differ from our prediction, as we only considered mismatches in the five nucleotides closest to the 3' end of the primer‐binding segment to infer amplification success, as they are considered the most disruptive (Stadhouders et al. 2010). However, some mismatches in the remaining primer sequence (Supp Table S3) may still influence amplification success if they affect primer binding, even if to a lesser extent than if they were located at the 3' end. Undocumented sequence variability in the primer‐binding sites may further affect predicted amplification success. In addition, other factors such as DNA quality play an important role. Indeed, in African wild dog, a species for which we predicted potential amplification issues due to the existence of two mismatches near the 3' end of the ATP6‐DR1 primer, variable amplification success was seen in field‐collected scat samples (data not shown), while amplification in the reference samples was successful.

Once sequences are obtained, identification of the corresponding carnivore species is dependent on the genetic distinctiveness among them and the availability of reliable reference sequences. While missing or inaccurate reference sequences have been consistently highlighted as a limitation of faecal mini‐barcoding approaches (Alberts et al. 2017; Chaves et al. 2012; Inoue and Akomo‐Okoue 2015; Rodríguez‐Castro et al. 2020), more and more sequences are becoming available thanks to studies such as this and international efforts such as the Vertebrate Genomes Project (Formenti et al. 2021). By the end of 2023, reference mitochondrial sequences for this *ATP6* mini‐barcode were available for 32 of the 42 reported terrestrial carnivores of mainland southern Africa. The addition of one new mitogenome assembly and two new mini‐barcode reference sequences from this study increased the number of identifiable carnivore species to 35 and reduced the average number of missing species to 3.2 per southern African country (Supp Table S1). While this is a significant coverage, the importance of comprehensive reference libraries cannot be stressed enough. All species lacking reference sequences are small carnivore species of the Herpestidae and Viverridae families, which are notoriously understudied, highlighting the importance of increased attention on these species. Sequence quality is also very important. For instance, the available mitogenome of the side‐striped jackal (KT448271) contained a large number of missing nucleotides for the sequence of the *ATP6* mini‐barcode and was dropped from our sequence comparisons; that said, we were able to replace it with our newly generated mini‐barcode sequence.

Lack of robust reference sequence databases can lead to the misidentification of a species or the inability to detect a species. We did not encounter such limitations with our analysis of previously published and newly generated *ATP6* mini‐barcode sequences of southern African terrestrial carnivores, which showed clear phylogenetic separation among all known species (Supp Figure S5). Furthermore, Inoue and Akomo‐Okoue (2015) reported flaws in databases due to intraspecific geographical differences in mini‐barcode sequences, making results less accurate. Indeed, this was the case in our study, as the banded mongoose samples could only be conclusively identified through comparison with local reference sequences, which differed from the previously published reference sequences by ten nucleotides, suggesting a deep divergence within this widely distributed species.

Our representative reference dataset (*n* = 61 sequences) is available for future research on Dryad (doi: 10.5061/dryad.bcc2fqzmt), and our marker assessment provides a tool for researchers, allowing them to evaluate how suited this mini‐barcode is for their project. We recommend that researchers consult our Supp Tables S1–S3 as part of their study design to determine how many species of interest are expected to be successfully detected in their study area using this *ATP6* mini‐barcode. For species of interest not covered by current reference sequences, we encourage researchers to contribute additional reference sequences to the scientific community (as we did for the Congo clawless otter) or to perform empirical amplification assays on reference samples to assess the risk of systematic failed amplification of that species. If a species of interest is predicted to amplify poorly with this mini‐barcode, we recommend relaxing PCR amplification conditions, trying different primers, or applying a different marker on samples that fail to amplify prior to considering them as ‘failed’.

As proof of principle, our in situ survey demonstrated the ability of the *ATP6* mini‐barcode to non‐invasively detect and identify carnivore species from field‐collected scat samples in a thornbush‐savannah environment in Namibia with high efficiency (94.9% species identification). While our survey provides a snapshot of carnivore presence, a more extensive effort across a broader area and longer duration is needed to fully capture the diversity and dynamics of the region's carnivore community. Even so, with minimal fieldwork effort (six teams walking transects for 2 h on 1 day in the dry season), 157 samples were collected and six species were identified. Those species included leopard, caracal, and black‐backed jackal, which, along with honey badger ( *Mellivora capensis* ), are considered indicators of mammalian species richness (Tshabalala et al. 2021).

The high species identification success (94.9%) was particularly remarkable as the samples for this study were collected in predominantly open habitat where they are exposed to harsh environmental conditions such as strong UV light (Mesa‐Cruz et al. 2016; Michalski et al. 2011); sample collection took place more than 13 years prior to sample processing; and storage conditions were –20°C, since –80°C freezers—the gold standard for faecal storage (Choo et al. 2015)—were not available. Species identification rates of samples stored for only a short time (Chaves et al. 2012; Gompper et al. 2006; Joshi et al. 2020; Khatoon et al. 2019) or preserved in ethanol (Akrim et al. 2018; Michalski et al. 2011; Rodríguez‐Castro et al. 2020) were comparable to those obtained in this study, demonstrating the robustness of this assay. The success of this mini‐barcode is likely largely attributable to the short amplicon length of only 172 base pairs, allowing it to amplify even in highly degraded faecal DNA (Hajibabaei et al. 2006; Meusnier et al. 2008) and would likely not have been achieved with full‐length barcodes. In addition, contrary to findings in a previous study (Gecchele et al. 2017), the arid climate of the study area likely minimised faecal DNA degradation attributed to humid environments and wet seasons (Brinkman et al. 2009; Michalski et al. 2011; Vynne et al. 2011).

The majority of samples (87.2%) were identified as black‐backed jackal. This likely reflects the relatively high abundance of this species in the study area (EIS Namibia 2022), compounded by a higher detection of jackal scat due to increased defaecation on roads for scent marking in canids (Hill et al. 2020; Stępniak et al. 2020). On the other hand, leopards, for instance, are more likely to use scraping and spraying for scent marking rather than defaecation (Rafiq et al. 2020), and species such as common genets are more likely to use latrines (Espírito‐Santo et al. 2007), while Afro‐asiatic wildcats are known to bury their faeces (Palmer and Fairall 1988). Consequently, canid species such as the black‐backed jackal are likely to be overrepresented in scat collections, while non‐canid species are more likely to be underrepresented or missed. To counter the effect of variable defaecation behaviour (e.g., species depositing faeces in locations that may be difficult to find or access) and large home ranges, collection strategies can be adapted to the species of interest and their respective defaecation behaviours (Gecchele et al. 2017; Gompper et al. 2006; Michalski et al. 2011). Nevertheless, five non‐canid species were detected with minimal field effort in our survey even in the absence of adaptive sample collection strategies, suggesting that the approach has great potential.

If increased species completeness is desired, sample collection can be scaled up with minimal effort. The main consideration to upscaling a mini‐barcoding‐based survey would be the increased cost associated with the laboratory work to process the additional samples. Camera‐trapping, on the other hand, has minimal cost associated with analysis, but expenses related to field effort are significantly higher than those of faecal mini‐barcoding. Camera‐traps are expensive and need to be deployed for prolonged periods to detect the full range of species, during which time maintenance checks are needed to replace batteries, memory cards, and potentially damaged or missing cameras. Thus, the budget for camera‐trapping field work requires funds to purchase equipment and regular visits to camera trap stations throughout the survey period (Gecchele et al. 2017; Janečka et al. 2011). Although detection of all species is ideal, very few field methods allow detection of all species present, and conservationists need to weigh the benefit of more complete data relative to increased sample collection effort and/or laboratory cost to decide on the ideal sample collection method and effort for their research question.

Detection approaches also have varying sensitivities for different species. Indeed, smaller species may be difficult to identify using camera trap photos, and species with similar appearances may be difficult to distinguish based on visual criteria alone, while approaches based on mini‐barcode identification have proven to be highly efficient. Detection methods also differ in their potential for downstream applications. For example, while camera‐trapping methods allow for visual evaluation of the animal and analyses of activity patterns, including on a temporal scale (since they record the exact time of detection), scat‐based methods provide a physical sample that can be further analysed to allow for dietary, parasitological, hormonal, and microbiome studies, which become much more powerful once samples have been identified at the species level (Schmidt‐Küntzel et al. 2018). Furthermore, DNA extracts obtained from scats can be used for additional research questions targeting genetic diversity, population structure as well as individual identification and parentage analyses (which allow assessments of social dynamics and dispersal patterns).

Even though this study was predominantly performed to demonstrate the applicability of the mini‐barcode in a field setting, it becomes evident that ecological/behavioural insights could be gleaned from larger datasets. The small dataset from our survey precludes extrapolation to other areas or species levels; however, we were able to observe statistically supported differences in defecation patterns that are compatible with known behavioural differences between species. Black‐backed jackals have been shown to both utilise and defaecate in multiple habitat types, distributing faeces approximately equally between open ground and bush cover (Humphries et al. 2016; Kamler et al. 2020), whereas other species such as leopards have been shown to rely on bush cover whilst hunting (Stander et al. 1997). This survey method thus has the potential to identify spatial defaecation behaviour of carnivores, as long as scat collection is carefully designed to cover a sufficient area and sample numbers to allow for extrapolation.

In conclusion, the efficiency of this *ATP6* mini‐barcode for species identification makes it an ideal tool for conservation research in sub‐Saharan Africa and likely other localities. Sample collection is fast and straightforward (157 samples collected by 6–12 people within 2 h). Laboratory work is basic and does not require any ‘next‐generation’ genetics equipment; it can therefore be performed in any fully equipped genetics laboratory in‐country, avoiding sample transport and permit logistics and their associated expenses. Species occurrence of common carnivores can be inferred reliably even with small sample numbers. For studies aiming for full species coverage, increasing sample size would lead to more representative results of species presence. Detection of carnivores, that have lower relative abundance or do not exhibit strong road‐marking behaviour, can further be increased through targeted sampling of habitats preferred by those species. While this mini‐barcode survey approach may not be suited for abundance estimates (unless a correction factor was established for each species or sample analysis was extended to individual‐level identification), it can provide valuable information on species diversity, defaecation behaviours, and spatial use by species for which sufficient samples are available, and thus contribute knowledge to inform conservation decisions as well as ongoing research.

## Author Contributions

**Amy Wong:** data curation (equal), formal analysis (lead), funding acquisition (equal), investigation (lead), visualization (equal), writing – original draft (lead), writing – review and editing (equal). **Eduardo Eizirik:** conceptualization (lead), data curation (equal), formal analysis (lead), visualization (equal), writing – original draft (equal), writing – review and editing (lead). **Klaus‐Peter Koepfli:** conceptualization (equal), data curation (equal), investigation (equal), writing – original draft (equal), writing – review and editing (lead). **Vera de Ferran:** formal analysis (lead), visualization (equal), writing – original draft (supporting), writing – review and editing (equal). **Tresia Shihepo:** data curation (equal), investigation (equal), writing – review and editing (supporting). **Anna Rose Lay:** methodology (equal), writing – review and editing (supporting). **Julia Zumbroich:** data curation (equal), writing – review and editing (supporting). **Nicola Rooney:** supervision (equal), writing – original draft (supporting), writing – review and editing (equal). **Laurie Marker:** conceptualization (supporting), funding acquisition (lead), resources (lead), writing – review and editing (supporting). **Anne Schmidt‐Küntzel:** conceptualization (lead), data curation (equal), formal analysis (equal), supervision (lead), visualization (equal), writing – original draft (lead), writing – review and editing (lead).

## Ethics Statement

Ethical approval was obtained from the University of Bristol's Animal Welfare and Ethical Review Body, reference code: UB/22/04. The research was performed under the research permit number AN202101032 of the Cheetah Conservation Fund (Namibian‐based Institute RCIV00122018).

## Conflicts of Interest

The authors declare no conflicts of interest.

## Supporting information


**Table S1.** Distribution of southern African terrestrial carnivores in southern Africa and subsaharan Africa.
**Table S2.** Overview of sequences included in the study.
**Table S3.** Predicted and empirical amplification success of the *ATP6* mini‐barcode in southern African terrestrial carnivore species.
**File S4.** Detailed information of laboratory work performed for the generation of additional *ATP6* reference sequences (see main text, ‘Building an *ATP6* sequence reference database’).
**Figure S5.** Phylogenetic relationships among *ATP6* mini‐barcode sequences reconstructed with the full curated dataset comprising 137 sequences. The sequences include previously existing GenBank submissions (identified by species name and accession number), sequences previously reported by Chaves et al. 2012 (identified by RefSeqB, species name, and local ID), as well as sequences generated as part of this study (identified as RefSeqC or RefSeqK, species name, and local ID). See Appendix 2 for details of all sequences. The phylogeny was estimated using a Neighbour‐joining algorithm and *p*‐distances, with nodal support assessed with 500 nonparametric bootstrap replicates.
**Figure S6.** Phylogenetic relationships among *ATP6* mini‐barcode sequences reconstructed with an alignment comprising 69 sequences, including the representative reference dataset (61 sequences) and additional sequences (identical haplotypes sampled in different individuals) from six species (*Parahyaena brunnea, Helogale parvula, Herpestes sanguineus, Felis lybica, Felis nigripes, Panthera pardus, Lycaon pictus, and Poecilogale albinucha*) included for visualization purposes. Sequence identifiers are the same as in Appendices 2 and 5. The phylogeny was estimated using a Neighbour‐joining algorithm and *p*‐distances, with nodal support assessed with 500 nonparametric bootstrap replicates.
**Figure S7.** Phylogenetic relationships among *ATP6* mini‐barcode sequences reconstructed with the same dataset as Appendix 6, comprising 69 sequences. Sequence identifiers are the same as in Appendices 2 and 5. The phylogeny was estimated using the Maximum Likelihood (ML) algorithm implemented in IQ‐TREE, with the HKY model of nucleotide substitution and gamma correction for rate variation among sites (alpha=0.3). Nodal support was assessed with 1000 ultrafast bootstrap replicates.
**Figure S8.** (A) Sample completeness curve. Rarefaction/extrapolation curve plotting relationship between the sample coverage and number of sampling units in edge and open habitat, Otjozondjupa region of Namibia. Sampling units refers to carnivore faecal samples. (B) Sample‐size‐based rarefaction and extrapolation sampling curve. Plotting the relationship between species coverage and the number of sampling units, in edge and open habitat, Otjozondjupa region of Namibia. Sampling units refers to carnivore faecal samples.

## Data Availability

Sequence data generated with the *ATP6* mini‐barcode primers (126 bp sequence, not including primer binding sites) were deposited on Dryad (doi: 10.5061/dryad.bcc2fqzmt; https://datadryad.org/stash/share/tQXDW_o7WE2wuW4vPq0QjE‐e9ATNnv04tYAalpLivqY); information on locality and samples is presented in Appendix S2. Sequence data generated with the primers amplifying a part of *ATP8* and *ATP6* were deposited on GenBank (see Appendix S2 for accession numbers). The mitogenome assembly of the Congo clawless otter was submitted to GenBank (accession number PV256478) as well as the same Dryad project as the newly generated reference sequences (doi: 10.5061/dryad.bcc2fqzmt; https://datadryad.org/stash/share/tQXDW_o7WE2wuW4vPq0QjE‐e9ATNnv04tYAalpLivqY). A sequence alignment for the representative reference dataset is available as a tool for species identification and was also submitted to the Dryad project (doi: 10.5061/dryad.bcc2fqzmt; https://datadryad.org/stash/share/tQXDW_o7WE2wuW4vPq0QjE‐e9ATNnv04tYAalpLivqY).
