# Supplementary material for: Identifying Cryptic Mammals With Non‐Invasive Methods: An Effective Molecular Species Identification Tool to Survey Southern African Terrestrial Carnivores
Source: Ecol Evol. 2025 Apr 21;15(4):e71223. doi: 10.1002/ece3.71223 (PMC12011410; doi:10.1002/ece3.71223)
Supplement: Supplementary file 1 — Table S1. Distribution of southern African terrestrial carnivores in southern Africa and subsaharan Africa. Table S2. Overview of sequences included in the study. Table S3. Predicted and empirical amplification success of the ATP6 mini‐barcode in southern African terrestrial carnivore species. File S4. Detailed information of laboratory work performed for the generation of additional ATP6 reference sequences (see main text, ‘Building an ATP6 sequence reference database’). Figure S5. Phylogenetic relationships among ATP6 mini‐barcode sequences reconstructed with the full curated dataset comprising 137 sequences. The sequences include previously existing GenBank submissions (identified by species name and accession number), sequences previously reported by Chaves et al. 2012 (identified by RefSeqB, species name, and local ID), as well as sequences generated as part of this study (identified as RefSeqC or RefSeqK, species name, and local ID). See Appendix 2 for details of all sequences. The phylogeny was estimated using a Neighbour‐joining algorithm and p‐distances, with nodal support assessed with 500 nonparametric bootstrap replicates. Figure S6. Phylogenetic relationships among ATP6 mini‐barcode sequences reconstructed with an alignment comprising 69 sequences, including the representative reference dataset (61 sequences) and additional sequences (identical haplotypes sampled in different individuals) from six species (Parahyaena brunnea, Helogale parvula, Herpestes sanguineus, Felis lybica, Felis nigripes, Panthera pardus, Lycaon pictus, and Poecilogale albinucha) included for visualization purposes. Sequence identifiers are the same as in Appendices 2 and 5. The phylogeny was estimated using a Neighbour‐joining algorithm and p‐distances, with nodal support assessed with 500 nonparametric bootstrap replicates. Figure S7. Phylogenetic relationships among ATP6 mini‐barcode sequences reconstructed with the same dataset as Appendix 6, comprising 69 sequences. Sequence [file ECE3-15-e71223-s001.zip › ece371223-sup-0001-FileS4.docx]

File S4:

Detailed information of laboratory work performed for the generation of additional *ATP6* reference sequences (see main text, ‘Building an *ATP6* sequence reference database’):

Cheetah Conservation Fund:
The species included were: *Crocuta crocuta, Parahyaena brunnea,* *Proteles cristata,* *Herpestes sanguineus,* *Mungos mungo*, *Genetta genetta*, *Acinonyx jubatus,* *Caracal caracal, Felis lybica,* *Leptailurus serval,* *Panthera leo,* *Panthera pardus,* *Lupulella mesomelas*, *Lycaon pictus,* *Otocyon megalotis,* *Vulpes chama,* *Ictonyx striatus*. 
For the 17 Namibian carnivore samples, PCR products were obtained as part of the marker assessment (see main text, ‘Verification of amplification success of the *ATP6* mini-barcode’). Sequencing reactions were carried out for both ATP6-DF3 and ATP6-DR1 on 1 μl purified PCR product in 10 μl reactions using BigDye™ Terminator v1.1 Cycle Sequencing Kits (Applied Biosystems™) according to the manufacturer's protocol. Sequencing products were cleaned with an EDTA/ethanol precipitation and run on a 3500 ABI Genetic Analyser (Applied Biosystems™). Base calling was carried out using Sequencing Analysis Software 7 (Applied Biosystems™), chromatograms visually verified and edited in Geneious Prime 2022.1.1 and 2024.0.3 (https://www.geneious.com), and consensus sequences created.

University of California, Los Angeles:
The species included were: *Genetta tigrina*, *Mungos mungo*, *Lupulella adusta*, *Lupulella mesomelas mesomelas* and *Lupulella mesomelas schmidti*, *Hydrictis maculicollis*, *Ictonyx striatus*, *Mellivora capensis*, and *Poecilogale albinucha*. 
For the nine carnivore species, genomic DNA was extracted from frozen whole blood or tissue samples collected from these species using either phenol-chloroform-isoamyl alcohol extraction followed by purification using ethanol precipitation and resuspension in TE (pH 8) buffer (Strauss, 1998) or the QIAamp DNA Mini Kit (Qiagen, Valencia, CA, USA) following the manufacturer’s instructions. A PCR master mix was set up and then aliquoted into individual tubes of 50 μl containing: 1 μL of DNA (∼0.1-0.5 μg), 5 μL 10x PCR buffer, 5 μL of 25mM MgCl2, 1 μL 10mM dNTP mix, 1 μL of 25 pM/uL forward and reverse primers (ATP6-DF1 and ATP6-DR1; Trigo et al. 2008), 0.3 μL Taq polymerase (Sigma-Aldrich, St. Louis, MO, USA), and 35.7 μL sterile double-distilled water. The segment was amplified in an MWG-Biotech Primus 96 Plus Thermal Cycler (Eurofins Genomics, Hunstville, AL, USA) with the thermal cycling conditions: one pre-denaturation cycle of 94 °C for 3 min followed by 30 cycles of 94 °C for 30 s, 52 °C for 30 s, 72 °C for 45 s; one cycle of 72 °C for 5 min; and a hold at 4 °C. A negative control (1 ul double-distilled water) was included with the PCRs. PCR products were visualized and checked in a 2% agarose/Tris-acetic acid-EDTA (TAE) gel with a 100 bp DNA ladder (Promega, Madison, WI, USA) run in 1x TAE buffer at 100 V for 60 min and stained with SYBR™ Safe DNA Gel Stain (Thermo-Fisher Scientific, Waltham, MA, USA). PCR products were then purified with Exonuclease I and Shrimp Alkaline Phosphatase according to the manufacturer’s instructions (Exo-SAP, Affymetrix, Santa Clara, CA, USA). Purified products were cycle sequenced in a 10 μL reaction mix containing either the ATP8-DF1 or ATP6-DR1 primers, the BigDye Terminator v3.1 Cycle Sequencing Kit (Life Technologies, Grand Island, NY, USA) and the SeqSaver Sequencing Premix Dilution Buffer (Sigma-Aldrich, St. Louis, MO, USA) with the following thermal cycling settings: one cycle at 96 °C for 1 min; 45 cycles at 96 °C for 10 sec, 50 °C for 5 sec, 60 °C for 4 min; and a hold at 4 °C. Cycle sequencing products were purified using EDTA/ethanol precipitation and shipped to the DNA Analysis Facility on Science Hill at Yale University (New Haven, CT, USA), where they were run on an Applied Biosystems 3130XL Genetic Analyzer (Life Technologies, Waltham, MA, USA). Chromatograms of both forward and reverse sequences were originally inspected and edited in Geneious Pro 4.7 and then rechecked using Geneious Prime 2023.2.1 ([https://www.geneious.com](http://www.geneious.com/)). Consensus sequences were created, and the segment corresponding to the *ATP6* mini-barcode was extracted to be used for sequence comparisons.

Strauss, W. M. (1998). Preparation of genomic DNA from mammalian tissue. *Current protocols in molecular biology*, *42*(1), 2-2.

Trigo, T. C., Freitas, T. R. O., Kunzler, G., Cardoso, L., Silva, J. C. R., Johnson, W. E., ... & Eizirik, E. (2008). Inter‐species hybridization among Neotropical cats of the genus Leopardus, and evidence for an introgressive hybrid zone between *L. geoffroyi* and *L. tigrinus* in southern Brazil. *Molecular Ecology*, *17*(19), 4317-4333.
