# Supplementary figures and images for: Identifying Cryptic Mammals With Non‐Invasive Methods: An Effective Molecular Species Identification Tool to Survey Southern African Terrestrial Carnivores
Source: Ecol Evol. 2025 Apr 21;15(4):e71223. doi: 10.1002/ece3.71223 (PMC12011410; doi:10.1002/ece3.71223)

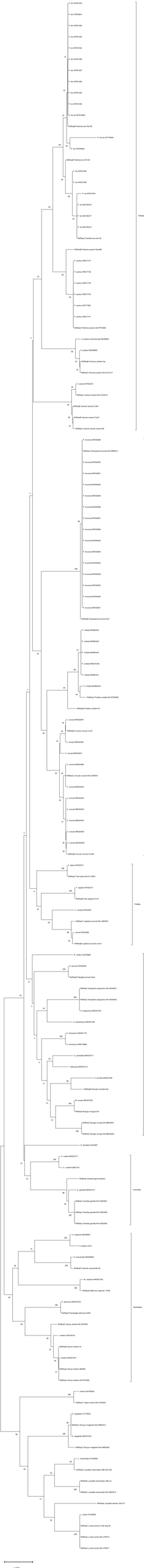

0.050

Supplement: Supplementary file 1 — Table S1. Distribution of southern African terrestrial carnivores in southern Africa and subsaharan Africa. Table S2. Overview of sequences included in the study. Table S3. Predicted and empirical amplification success of the ATP6 mini‐barcode in southern African terrestrial carnivore species. File S4. Detailed information of laboratory work performed for the generation of additional ATP6 reference sequences (see main text, ‘Building an ATP6 sequence reference database’). Figure S5. Phylogenetic relationships among ATP6 mini‐barcode sequences reconstructed with the full curated dataset comprising 137 sequences. The sequences include previously existing GenBank submissions (identified by species name and accession number), sequences previously reported by Chaves et al. 2012 (identified by RefSeqB, species name, and local ID), as well as sequences generated as part of this study (identified as RefSeqC or RefSeqK, species name, and local ID). See Appendix 2 for details of all sequences. The phylogeny was estimated using a Neighbour‐joining algorithm and p‐distances, with nodal support assessed with 500 nonparametric bootstrap replicates. Figure S6. Phylogenetic relationships among ATP6 mini‐barcode sequences reconstructed with an alignment comprising 69 sequences, including the representative reference dataset (61 sequences) and additional sequences (identical haplotypes sampled in different individuals) from six species (Parahyaena brunnea, Helogale parvula, Herpestes sanguineus, Felis lybica, Felis nigripes, Panthera pardus, Lycaon pictus, and Poecilogale albinucha) included for visualization purposes. Sequence identifiers are the same as in Appendices 2 and 5. The phylogeny was estimated using a Neighbour‐joining algorithm and p‐distances, with nodal support assessed with 500 nonparametric bootstrap replicates. Figure S7. Phylogenetic relationships among ATP6 mini‐barcode sequences reconstructed with the same dataset as Appendix 6, comprising 69 sequences. Sequence [file ECE3-15-e71223-s001.zip › ece371223-sup-0001-FigureS5.pdf]

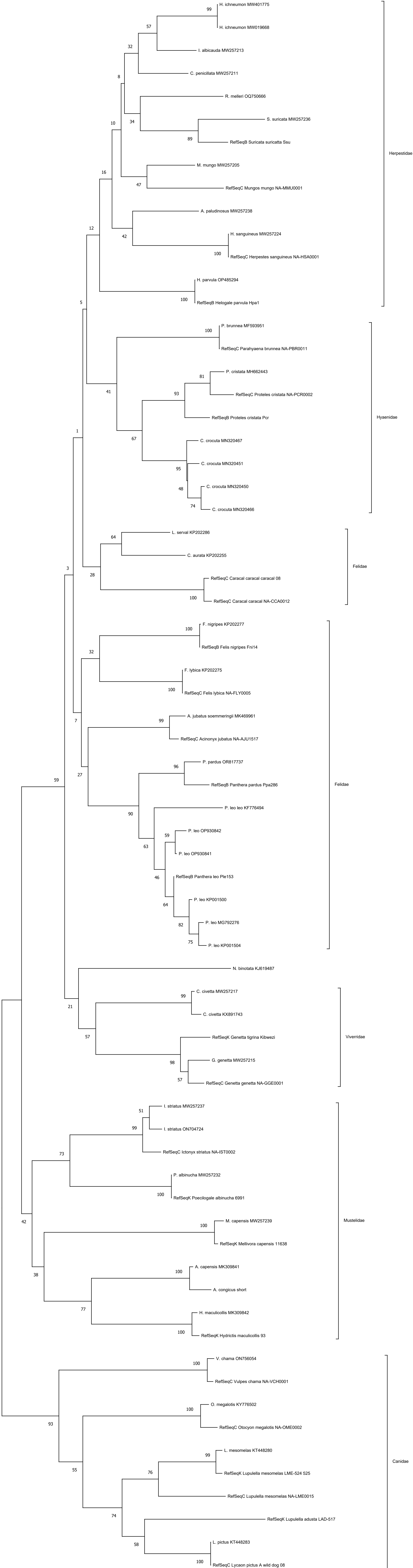

0,050

Supplement: Supplementary file 1 — Table S1. Distribution of southern African terrestrial carnivores in southern Africa and subsaharan Africa. Table S2. Overview of sequences included in the study. Table S3. Predicted and empirical amplification success of the ATP6 mini‐barcode in southern African terrestrial carnivore species. File S4. Detailed information of laboratory work performed for the generation of additional ATP6 reference sequences (see main text, ‘Building an ATP6 sequence reference database’). Figure S5. Phylogenetic relationships among ATP6 mini‐barcode sequences reconstructed with the full curated dataset comprising 137 sequences. The sequences include previously existing GenBank submissions (identified by species name and accession number), sequences previously reported by Chaves et al. 2012 (identified by RefSeqB, species name, and local ID), as well as sequences generated as part of this study (identified as RefSeqC or RefSeqK, species name, and local ID). See Appendix 2 for details of all sequences. The phylogeny was estimated using a Neighbour‐joining algorithm and p‐distances, with nodal support assessed with 500 nonparametric bootstrap replicates. Figure S6. Phylogenetic relationships among ATP6 mini‐barcode sequences reconstructed with an alignment comprising 69 sequences, including the representative reference dataset (61 sequences) and additional sequences (identical haplotypes sampled in different individuals) from six species (Parahyaena brunnea, Helogale parvula, Herpestes sanguineus, Felis lybica, Felis nigripes, Panthera pardus, Lycaon pictus, and Poecilogale albinucha) included for visualization purposes. Sequence identifiers are the same as in Appendices 2 and 5. The phylogeny was estimated using a Neighbour‐joining algorithm and p‐distances, with nodal support assessed with 500 nonparametric bootstrap replicates. Figure S7. Phylogenetic relationships among ATP6 mini‐barcode sequences reconstructed with the same dataset as Appendix 6, comprising 69 sequences. Sequence [file ECE3-15-e71223-s001.zip › ece371223-sup-0001-FigureS6.pdf]

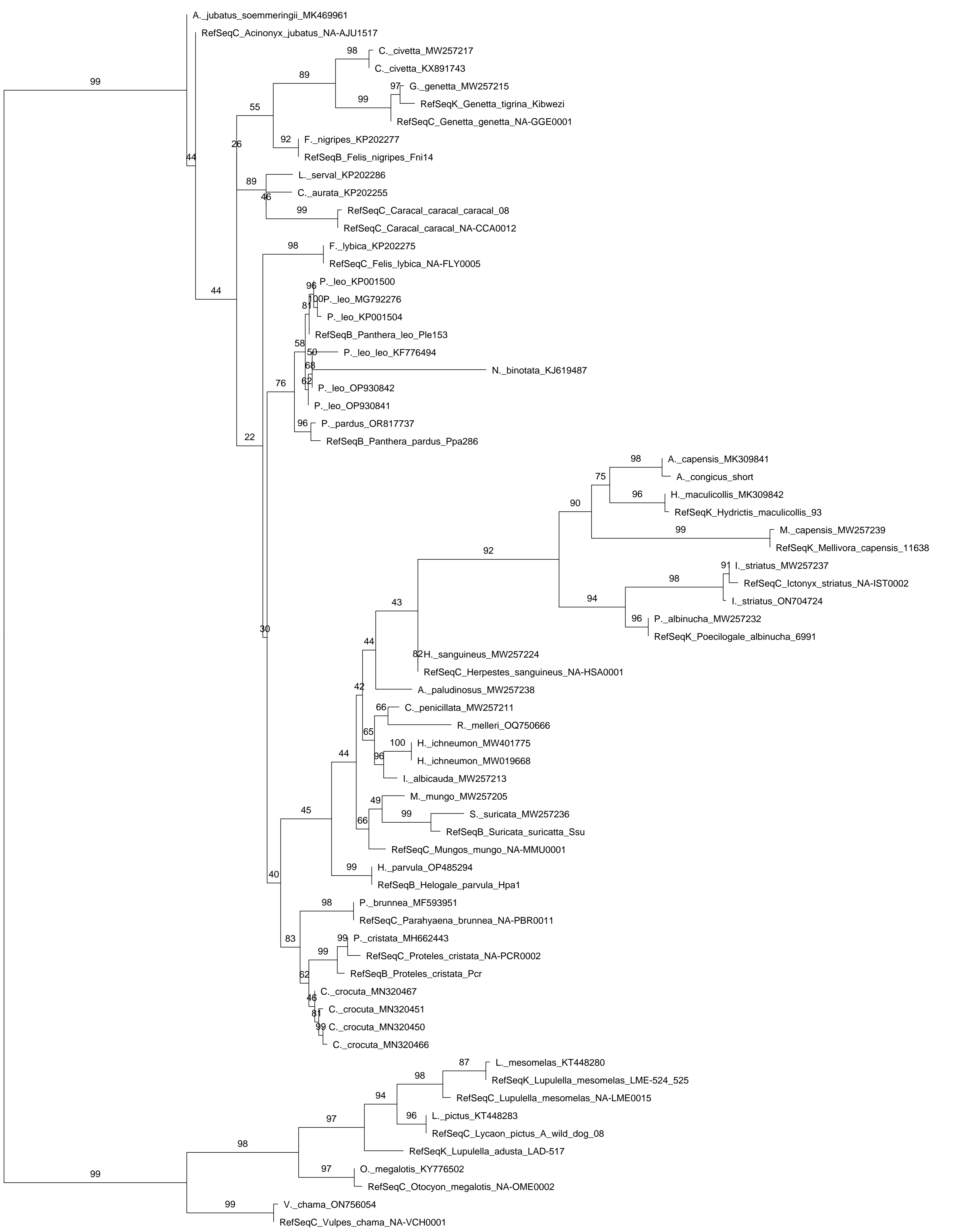

Supplement: Supplementary file 1 — Table S1. Distribution of southern African terrestrial carnivores in southern Africa and subsaharan Africa. Table S2. Overview of sequences included in the study. Table S3. Predicted and empirical amplification success of the ATP6 mini‐barcode in southern African terrestrial carnivore species. File S4. Detailed information of laboratory work performed for the generation of additional ATP6 reference sequences (see main text, ‘Building an ATP6 sequence reference database’). Figure S5. Phylogenetic relationships among ATP6 mini‐barcode sequences reconstructed with the full curated dataset comprising 137 sequences. The sequences include previously existing GenBank submissions (identified by species name and accession number), sequences previously reported by Chaves et al. 2012 (identified by RefSeqB, species name, and local ID), as well as sequences generated as part of this study (identified as RefSeqC or RefSeqK, species name, and local ID). See Appendix 2 for details of all sequences. The phylogeny was estimated using a Neighbour‐joining algorithm and p‐distances, with nodal support assessed with 500 nonparametric bootstrap replicates. Figure S6. Phylogenetic relationships among ATP6 mini‐barcode sequences reconstructed with an alignment comprising 69 sequences, including the representative reference dataset (61 sequences) and additional sequences (identical haplotypes sampled in different individuals) from six species (Parahyaena brunnea, Helogale parvula, Herpestes sanguineus, Felis lybica, Felis nigripes, Panthera pardus, Lycaon pictus, and Poecilogale albinucha) included for visualization purposes. Sequence identifiers are the same as in Appendices 2 and 5. The phylogeny was estimated using a Neighbour‐joining algorithm and p‐distances, with nodal support assessed with 500 nonparametric bootstrap replicates. Figure S7. Phylogenetic relationships among ATP6 mini‐barcode sequences reconstructed with the same dataset as Appendix 6, comprising 69 sequences. Sequence [file ECE3-15-e71223-s001.zip › ece371223-sup-0001-FigureS7.pdf]

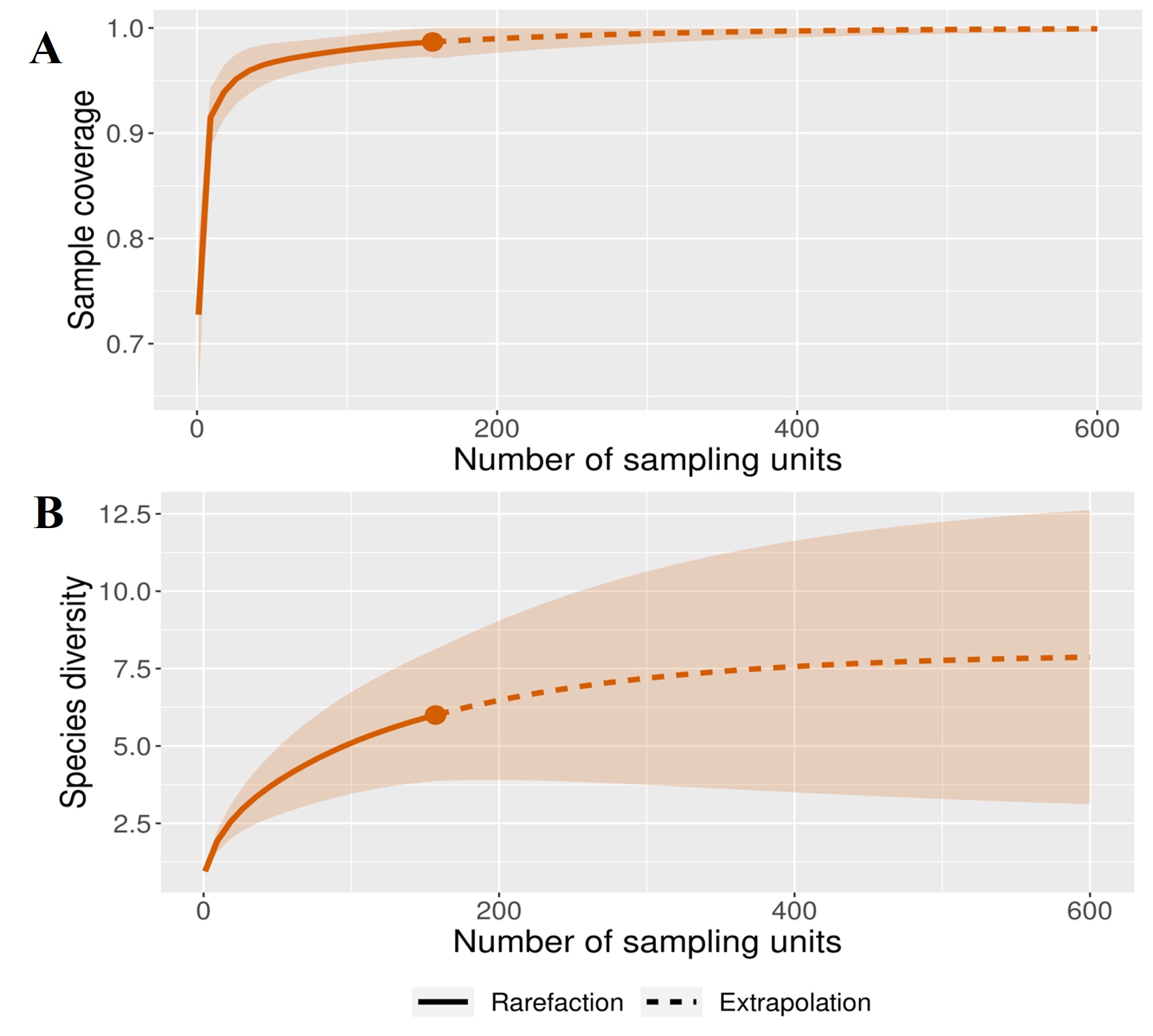

Supplement: Supplementary file 1 — Table S1. Distribution of southern African terrestrial carnivores in southern Africa and subsaharan Africa. Table S2. Overview of sequences included in the study. Table S3. Predicted and empirical amplification success of the ATP6 mini‐barcode in southern African terrestrial carnivore species. File S4. Detailed information of laboratory work performed for the generation of additional ATP6 reference sequences (see main text, ‘Building an ATP6 sequence reference database’). Figure S5. Phylogenetic relationships among ATP6 mini‐barcode sequences reconstructed with the full curated dataset comprising 137 sequences. The sequences include previously existing GenBank submissions (identified by species name and accession number), sequences previously reported by Chaves et al. 2012 (identified by RefSeqB, species name, and local ID), as well as sequences generated as part of this study (identified as RefSeqC or RefSeqK, species name, and local ID). See Appendix 2 for details of all sequences. The phylogeny was estimated using a Neighbour‐joining algorithm and p‐distances, with nodal support assessed with 500 nonparametric bootstrap replicates. Figure S6. Phylogenetic relationships among ATP6 mini‐barcode sequences reconstructed with an alignment comprising 69 sequences, including the representative reference dataset (61 sequences) and additional sequences (identical haplotypes sampled in different individuals) from six species (Parahyaena brunnea, Helogale parvula, Herpestes sanguineus, Felis lybica, Felis nigripes, Panthera pardus, Lycaon pictus, and Poecilogale albinucha) included for visualization purposes. Sequence identifiers are the same as in Appendices 2 and 5. The phylogeny was estimated using a Neighbour‐joining algorithm and p‐distances, with nodal support assessed with 500 nonparametric bootstrap replicates. Figure S7. Phylogenetic relationships among ATP6 mini‐barcode sequences reconstructed with the same dataset as Appendix 6, comprising 69 sequences. Sequence [file ECE3-15-e71223-s001.zip › ece371223-sup-0001-FigureS8.jpg]
